# Aspects of Uniform Horizontal Magnetic Field and Nanoparticle Aggregation in the Flow of Nanofluid with Melting Heat Transfer

**DOI:** 10.3390/nano12061000

**Published:** 2022-03-18

**Authors:** Fuzhang Wang, Rangaswamy Naveen Kumar, Ballajja C. Prasannakumara, Umair Khan, Aurang Zaib, Abdel-Haleem Abdel-Aty, Ibrahim S. Yahia, Mohammed S. Alqahtani, Ahmed M. Galal

**Affiliations:** 1 School of Mathematical and Statistics, Xuzhou University of Technology, Xuzhou 221018, China; wangfuzhang1984@163.com; 2 Department of Mathematics, Nanchang Institute of Technology, Nanchang 330044, China; 3 Department of Mathematics, Davangere University, Shivagangotri, Davangere 577002, Karnataka, India; nkrmaths@gmail.com (R.N.K.); dr.bcprasanna@gmail.com (B.C.P.); 4 Department of Mathematical Sciences, Faculty of Science and Technology, Universiti Kebangsaan Malaysia, Bangi 43600, Selangor, Malaysia; umairkhan@iba-suk.edu.pk; 5 Department of Mathematics and Social Sciences, Sukkur IBA University, Sukkur 65200, Sindh, Pakistan; 6 Department of Mathematical Sciences, Federal Urdu University of Arts, Science & Technology, Gulshan-e-Iqbal, Karachi 75300, Sindh, Pakistan; 7 Department of Physics, College of Sciences, University of Bisha, Bisha 61922, Saudi Arabia; amabdelaty@ub.edu.sa; 8 Physics Department, Faculty of Science, Al-Azhar University, Assiut 71524, Egypt; 9 Laboratory of Nano-Smart Materials for Science and Technology (LNSMST), Department of Physics, Faculty of Science, King Khalid University, Abha 61413, Saudi Arabia; isyahia@gmail.com; 10 Research Center for Advanced Materials Science (RCAMS), King Khalid University, Abha 61413, Saudi Arabia; 11 Nanoscience Laboratory for Environmental and Biomedical Applications (NLEBA), Metallurgical Lab. 1, Department of Physics, Faculty of Education, Ain Shams University, Roxy, Cairo 11757, Egypt; 12 Radiological Sciences Department, College of Applied Medical Sciences, King Khalid University, Abha 61421, Saudi Arabia; mosalqhtani@kku.edu.sa; 13 BioImaging Unit, Space Research Centre, Michael Atiyah Building, University of Leicester, Leicester LE1 7RH, UK; 14 Mechanical Engineering Department, College of Engineering, Prince Sattam Bin Abdulaziz University, Wadiad Dawaser 11991, Saudi Arabia; ahm.mohamed@psau.edu.sa; 15 Production Engineering and Mechanical Design Department, Faculty of Engineering, Mansoura University, Mansoura 35516, Egypt

**Keywords:** nanoparticle aggregation, uniform horizontal magnetic field, thermal radiation, melting effect, homogeneous and heterogeneous chemical reactions, rotating disk

## Abstract

The current exploration focuses on the impact of homogeneous and heterogeneous chemical reactions on titanium dioxide-ethylene glycol (EG)-based nanoliquid flow over a rotating disk with thermal radiation. In this paper, a horizontal uniform magnetic field is used to regularise the flow field produced by a rotating disk. Further, we conduct a comparative study on fluid flow with and without aggregation. Suitable transformations are used to convert the governing partial differential equations (PDEs) into ordinary differential equations (ODEs). Later, the attained system is solved numerically by means of the shooting method in conjunction with the Runge–Kutta–Fehlberg fourth-fifth-order method (RKF-45). The outcome reveals that the fluid flow without nanoparticle aggregation shows enhanced heat transport than for augmented values of melting parameter. Furthermore, for augmented values of strength of homogeneous and heterogeneous reaction parameters, the mass transfer is greater in fluid flow with aggregation conditions.

## 1. Introduction

Nanofluids, which are made up of solid nanoparticles (NPs) that are roughly 1–100 nm in size, have attracted a lot of interest as they are believed to have better qualities than traditional heat-transfer liquids. All materials whose dimensions are less than 100 nanometres (nm) are referred to as NPs. In the early 1990s, Choi proposed the notion of nanoliquids for the first time. NPs with a significantly bigger surface area and smaller size have the potential to even further enhance heat-transfer capabilities and liquid stability. Scientists are also interested in TiO2-based materials because of their potential and success in a variety of fields, including photocatalysis for self-cleaning of solid surfaces, fillers, memory device sensors, solar cells, and water purification. Several works [1,2,3,4,5,6,7] have recently studied the various nanoliquid flows via different surfaces. Both rheological and thermal characteristics are influenced by NP aggregations. One of the most important parameters governing nanoparticle aggregation is surface charge. As a result, aggregation is a critical aspect in any nanofluid’s thermal applications. To provide a reduced thermal-resistance route, the aggregated NPs prefer to form percolating networks and linear chains. Consequently, heat may be transported extremely quickly through the clusters, which, together with the increased effective aggregation volume compared to NPs, can improve the nanofluid’s thermal conductivity. Ellahi [8] investigated the effects of aggregation on a water-based alumina nanofluid that was passed through a permeable wedge. In a carbon nanotube water-based nanofluid, Benos et al. [9] investigated the critical role of aggregations. Karvelas et al. [10] swotted the magnetic aggregation of iron-oxide NPs. Mahanthesh et al. [11] swotted the heat transport of NPs via NP aggregation. By studying NP aggregation, Mackolil and Mahanthesh [12] revealed the upshot of magnetic fields on the convective flow of nanomaterial liquid. By addressing the shape and NP aggregation, Motlagh and Kalteh [13] investigated heat transfer in a nanochannel. The leverage of Joule heating and NP aggregation on the nanofluid flow was studied by Swain and Mahanthesh [14]. Sabu et al. [15] studied the kinetics of nanoparticle aggregation in a convective nanomaterial flow travelling across an inclined flat plate.

Due to its practical and theoretical significance, the study of heat transport and liquid flow across a rotating disk is regarded one of the most important subjects in fluid mechanics. In numerous forms of equipment, such as gas turbines and computer disc drives, heat transmission throughout the spinning body has significant repercussions. Due to its practical implications in many applications, the examination of boundary-layer streams spanning diverse disk movements has received a lot of attention in recent years. Turkyilmazoglu [16,17] studied the two- and three-dimensional fluid flow and heat transport caused by a spinning stretching disk. Kumar et al. [18] conducted a comparative study on the nanofluid stream on a permeable disk. The Smoluchowski temperature slip was considered by khan et al. [19] to educe the slipstream of non-Newtonian fluid across revolving-disk Maxwell velocity-slip conditions. Shoaib et al. [20] explored the flow of a nanofluidic system of Ree–Eyring fluid across a disk.

The process of heat transport will be stable if heat is supplied efficiently, transformed from one area to another, and regulated. Heat is transported owing to a variety of factors such as non-uniform heat source/sink, and so on. To describe the heat-transmission properties, a thermal equation is constructed in the present article by incorporating thermal radiation. Makinde [21] used radiation and internal heat production to study the hydromagnetic convection flow towards an upright plate contained in a porous material. Archanaet al. [22] used the features of radiation and slip effect to confer a Falkner–Skan stream of Casson nanoliquid on a wedge. Garia et al. [23] conferred the consequence of non-Fourier heat flux on radiative flow through two different geometries. Rawat et al. [24] investigated the radiative flow of nanofluid with varied parameters using cone and wedge geometries. Yaseen et al. [25] used the characteristics of the radiation effect to confer radiative stream of kerosene oil-based nanoliquid flow between rotating disks. Several chemically reactive systems, such as burning, biochemical frameworks, and catalysis, contain homogeneous-heterogeneous (H-H) reactions. The relationship among H-H reactions is chiefly perplexing. With the exception of the existence of a catalyst, a proportion of reactions may proceed slowly. Khan et al. [26] studied H-H reactions in the Sutterby fluid stream on a rotating disc. Gowda et al. [27] studied the upshot of H-H reactions on nanoliquid flow over a poignant disk. Christopher et al. [28] studied the impact of H-H reactions on a nanoliquid flow suspended with alumina and copper NPs over a surface. Abbas et al. [29] examined the properties of H-H reactions occurring in a fluid stream travelling through a spinning disc. Sunthrayuth et al. [30] conveyed the stream of a nanofluid via a melting surface with H-H reactions and performed a comparative study on fluid flow with and without NP aggregation.

Melting heat transfer is a fascinating sub discipline of thermodynamics. Geothermal energy recovery, heat engines, thermocouples, permafrost melting, silicon-wafer manufacturing, heat exchangers, and hot extrusion are all applications of the melting phenomena. In its most basic form, the melting heat-transference issue is a boundary problem that requires the employment of strong computer algorithms to solve. However, another tactic is to emphasise the boundary-layer stream in melting phase-change problems, where the melting phenomena may be represented as a boundary constraint. Mabood et al. [31] explained the melting heat transmission and radiative stream of a hybrid nanoliquid beyond a stretchable shape. Radhika et al. [32] studied the dust particle suspension on the flow of a fluid containing many nanoparticles by taking the melting heat transferal event into account. Reddy et al. [33] studied the melting heat transfer properties of a nanoliquid using a sheet that is stretching at a uniform pace. Mallikarjuna et al. [34] studiedthe melting heat transfer in a dusty nanoliquid passing via a porous stretchable surface. Khan et al. [35] studied the melting effect and radiation heat transferal on a nanofluid stream passing through a Riga plate.

In the above-mentioned articles, nanoliquid flow over a stretching rotating disk with melting effect and NP aggregation was not yet discussed to the best of the authors’ knowledge. As it is well-acknowledged, there are a number of approaches that are available to provide a few appropriate solutions to this kind of issue. There has never been a numerical solution for the specified flow. This research gap prompted researchers to employ a numerical technique (RKF-45) and a shooting strategy to study the upshot of effective parameters on the flow characteristics of ethylene glycol-based nanofluid. Further, we have carried out a comparative study on liquid flow with and without aggregation. Finally, the aim of the study is to answer the following research questions:

- To explore the fluid flow, heat, and mass-transfer behaviour with and without NP aggregation.
- What effect do different dimensionless factors have on the flow, heat, and mass-transport behaviour of nanofluids?

## 2. Mathematical Formulation

**Figure 1:**
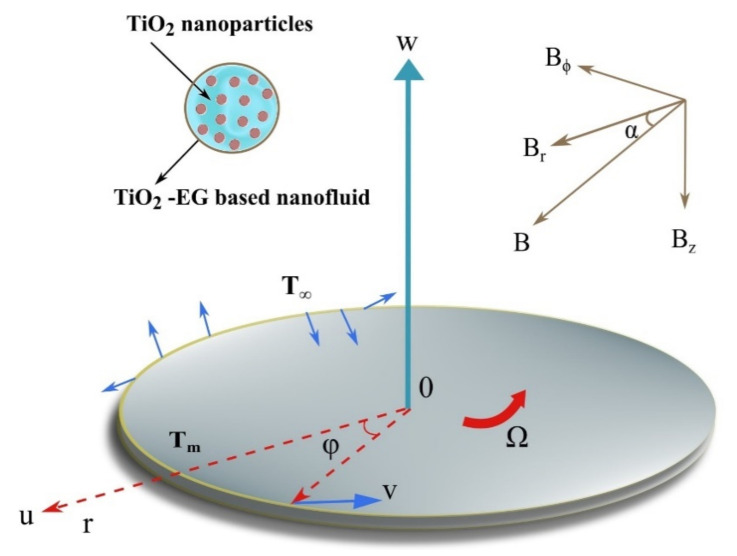
Flow geometry representing the flow of TiO_2_-ethylene glycol-based nanofluid over a disk rotating about its axial direction z-axis with a constant angular speed Ω and uniform horizontal magnetic field.

Consider a steady incompressible flow of TiO_2_-ethylene glycol-based nanofluid over a disk rotating about its axial axis z with a constant angular speed Ω. Let, V=(u,v,w) as the induced velocity field. The physical phenomenon is explained with the help of Figure 1. The uniform external magnetic field of strength B applied to a disk. In the cylindrical coordinate system, it can be written as (see Turkyilmazoglu [36]):

(1) $$B=\left(B_{r},B_{\varphi },B_{z}\right).$$

Due to the conducting disk, the current density induced can be represented by (see Turkyilmazoglu [36]):

(2) $$J=\sigma \left(E+V\times B\right),$$

Since $\nabla \times E=-\frac{\partial B}{\partial t}=0$, the electric field E can be taken as zero due to no polarisation. Further, it is assumed that the Re of the fluid is far greater than the magnetic Re (see Turkyilmazoglu [16,17]). Hence, no induced magnetic field takes place. As a result, the Lorentz force acting over the fluid flow due to the magnetic field can be written as (see Turkyilmazoglu [36]):

(3) $$J\times B=\sigma \left(V\times B\right)\times B.$$

The modified Krieger and Dougherty viscosity model and the Bruggeman thermal conductivity model are used to simulate nanoliquids with NP aggregation. In the presence of thermal radiation, the heat-transfer analysis is also taken into consideration. In addition, the melting effect and H-H reactions are taken into account. We suppose that the melting surface temperature is lower than the ambient temperature $\left(T_{m}<T_{\infty }\right)$. Flow analysis is accomplished with H-H reactions concerning dual chemical species $A^{*}$ and $B^{*}$. The model suggested by Chaudhary and Merkin [37,38]’s H-H reactions is assumed in the current study. For cubic autocatalysis, homogeneous reaction is as follows:

(4) $$A^{*}+2B^{*}\to 3B^{*},\;rate=k_{c}ab^{2}$$

(5) $$A^{*}\to B^{*},\;rate=k_{s}a$$

Moreover, these reactions are assumed to be isothermal.

The flow affected by the horizontal magnetic field, and all the above assumptions of the governing equations in the cylindrical coordinate system, lead to the following expressions (see Mackolil and Mahanthesh [12], Khan et al. [26] and Turkyilmazoglu [36]):

(6) ∇.V=0,

(7) $$(V.\nabla )V=\nu_{nf}\nabla^{2}V+\frac{1}{\rho_{nf}}J\times B,$$

(8) $$(V.\nabla )T=\alpha_{nf}\nabla^{2}T-\frac{1}{{\left(\rho C_{p}\right)}_{nf}}\nabla .q_{r},$$

where $q_{r}$ represents the radiative heat flux. Using Rosseland approximation (see Jain and Bohra [39]) $q_{r}$ is expressed as follows:

(9) $$q_{r}=-\frac{4\sigma^{*}}{3k^{*}}\frac{\partial T^{4}}{\partial z}.$$

Expansion of $T^{4}$ using Taylor’s series and neglecting higher-order terms, we have

(10) $$T^{4}≅4T_{\infty }^{3}T-3T_{\infty }^{4}$$

Now, by using Equations (9) and (10), in Equation (8) we obtain

(11) $$u\frac{\partial T}{\partial r}+w\frac{\partial T}{\partial z}=\alpha_{nf}\left(\frac{\partial^{2}T}{\partial r^{2}}+\frac{1}{r}\frac{\partial T}{\partial r}+\frac{\partial^{2}T}{\partial z^{2}}\right)+\frac{16\sigma^{*}T_{\infty }^{3}}{{\left(\rho C_{p}\right)}_{nf}3k^{*}}\left(\frac{\partial^{2}T}{\partial r^{2}}+\frac{1}{r}\frac{\partial T}{\partial r}+\frac{\partial^{2}T}{\partial z^{2}}\right),$$

(12) $$u\frac{\partial a}{\partial r}+w\frac{\partial a}{\partial z}=D_{A}\left(\frac{\partial^{2}a}{\partial r^{2}}+\frac{1}{r}\frac{\partial a}{\partial r}+\frac{\partial^{2}a}{\partial z^{2}}\right)-k_{c}ab^{2},$$

(13) $$u\frac{\partial b}{\partial r}+w\frac{\partial b}{\partial z}=D_{B}\left(\frac{\partial^{2}b}{\partial r^{2}}+\frac{1}{r}\frac{\partial b}{\partial r}+\frac{\partial^{2}b}{\partial z^{2}}\right)+k_{c}ab^{2}.$$

The essential boundary conditions for the proposed work are (see Khan et al. [26] Sunthrayuth et al. [30] and Turkyilmazoglu [36]):

(14) $$\left.\begin{array}{l} u=C_{0}r=U_{w}(r),v=\Omega r,k_{nf}\frac{\partial T}{\partial z}=\rho_{nf}\left(C_{s}\left(T_{m}-T_{0}\right)+\lambda \right)w,T=T_{m},\\ D_{A}\frac{\partial a}{\partial z}=k_{s}a,D_{B}\frac{\partial b}{\partial z}=-k_{s}a\;\mathrm{at}\;z=0,\\ u\to 0,v\to 0,w\to 0,T\to T_{\infty },a\to a_{0},b\to 0\;\mathrm{as}\;z\to \infty . \end{array}\right\}$$

### 2.1. Thermophysical Properties for Aggregation Approach

Based on experimental data, nanoliquids are known to have a high thermal conductivity. Additionally, erratic NP mobility or NP aggregation resulting in percolation activity may be exploited to enhance the thermal properties. Brownian randomness deteriorates when contrasted to aggregation, which increases aggregate mass, yet aggregate percolation behaviour may boost heat conductivity. As a consequence, for NP aggregation, the effective viscosity, density, heat capacitance, and thermal conductivity of nanofluid are as follows (see Refs. [8,9,40]):

(15) $$\mu_{nf}=\mu_{f}{\left(1-\frac{\phi_{agg}}{\phi_{\max}}\right)}^{-2.5*\phi_{\max}},$$

(16) $$\rho_{nf}=\left(1-\phi_{agg}\right)\rho_{f}+{\left(\phi \rho \right)}_{agg},$$

(17) $${\left(\rho C_{p}\right)}_{nf}=\left(1-\phi_{agg}\right){\left(\rho C_{p}\right)}_{f}+\phi_{agg}{\left(\rho C_{p}\right)}_{agg},$$

(18) $$k_{nf}=k_{f}\left(\frac{k_{agg}+2k_{f}+2\phi_{agg}\left(k_{agg}-k_{f}\right)}{k_{agg}+2k_{f}-\phi_{agg}\left(k_{agg}-k_{f}\right)}\right),$$

(19) $$\sigma_{nf}=\left(1+\frac{3\left(\frac{\sigma_{s}}{\sigma_{f}}-1\right)\phi_{agg}}{\left(\frac{\sigma_{s}}{\sigma_{f}}+2\right)-\left(\frac{\sigma_{s}}{\sigma_{f}}-1\right)\phi_{agg}}\right)\sigma_{f}.$$

### 2.2. Thermal Characteristics of Particles Aggregation

The modified Krieger and Dougherty model with the modified Maxwell model are used to calculate effective viscosity and thermal conductivity, respectively (see Refs. [8,9,40]):

(20) $$\phi_{agg}=\frac{\phi }{\phi_{\mathrm{int}}},\phi_{\mathrm{int}}={\left(\frac{R_{agg}}{R_{p}}\right)}^{D-3}$$

(21) $$\rho_{agg}=\left(1-\phi_{\mathrm{int}}\right)\rho_{f}+\phi_{\mathrm{int}}\rho_{s}$$

(22) $${\left(\rho C_{p}\right)}_{agg}=\left(1-\phi_{\mathrm{int}}\right){\left(\rho C_{p}\right)}_{f}+\phi_{\mathrm{int}}{\left(\rho C_{p}\right)}_{s}$$

(23) $$k_{agg}=\frac{k_{f}}{4}\left(\begin{array}{l} \left[3\phi_{\mathrm{int}}-1\right]\frac{k_{s}}{k_{f}}+\left[3\left(1-\phi_{\mathrm{int}}\right)-1\right]+\\ {\left[{\left[\left(3\phi_{\mathrm{int}}-1\right)\frac{k_{s}}{k_{f}}+\left(3\left(1-\phi_{\mathrm{int}}\right)-1\right)\right]}^{2}+\frac{8k_{s}}{k_{f}}\right]}^{\frac{1}{2}} \end{array}\right)$$

The maximum particle-packing fraction for spherical shape $\phi_{\max}$ is considered as 0.605. From the fractal theory, radii of aggregates $R_{agg}$ and radii of primary NPs $R_{p}$ (the value of $\frac{R_{agg}}{R_{p}}$ is considered as 3.34). For spherical shape, the fractal index D=1.8.

### 2.3. Similarity Transformations

To further ease the analysis of the problem, the following similarity variables transform the governing equations into the nondimensional form (see Turkyilmazoglu [17], Khan et al. [26] and Turkyilmazoglu [36]):

(24) $$\left.\begin{array}{l} u=r\Omega F(\eta ),v=r\Omega G(\eta ),w=\sqrt{\nu_{f}\Omega }H(\eta ),\eta =\sqrt{\frac{\Omega }{\nu_{f}}}z,\\ \theta (\eta )=\frac{T-T_{m}}{T_{\infty }-T_{m}},a=a_{0}\chi (\eta ),b=a_{0}\vartheta (\eta ). \end{array}\right\}$$

The Lorentz force can be decomposed into its components and expressed as (see Turkyilmazoglu [26]):

(25) $$J\times B=\sigma \left(-B_{\varphi }\left(B_{\varphi }u-B_{r}v\right),-B_{r}\left(B_{\varphi }u-B_{r}v\right),-\left(B_{\varphi }^{2}+B_{r}^{2}\right)w\right),$$

If the angle of inclination between the r-direction and the direction of the magnetic field vector $B=\left(B_{r},B_{\varphi },0\right)$ is defined as α; it can be written as (see Turkyilmazoglu [26]):

(26) $$B_{\varphi }=\left|B\right|\sin\alpha ,B_{r}=\left|B\right|\cos\alpha .$$

The following set of nondimensionless nonlinear differential equations is obtained:

(27) $$H^{\prime }+2F=0,$$

(28) $$\varepsilon_{1}F^{\prime \prime }-HF^{\prime }-F^{2}+G^{2}-\varepsilon_{2}\frac{\sigma_{nf}}{\sigma_{f}}M\sin\alpha \left[\sin\alpha F-\cos\alpha G\right]=0,$$

(29) $$\varepsilon_{1}G^{\prime \prime }-2FG-HG^{\prime }+\varepsilon_{2}\frac{\sigma_{nf}}{\sigma_{f}}M\cos\alpha \left[\sin\alpha F-\cos\alpha G\right]=0,$$

(30) $$\varepsilon_{1}H^{\prime \prime }-HH^{\prime }-\varepsilon_{2}\frac{\sigma_{nf}}{\sigma_{f}}MH=0,$$

(31) $$\frac{1}{\mathrm{Pr}}\varepsilon_{3}\left(\frac{k_{nf}}{k_{f}}+R\right)\theta^{\prime \prime }-H\theta^{\prime }=0,$$

(32) $$\frac{1}{Sc}\chi^{\prime \prime }-H\chi^{\prime }-k_{1}\chi \vartheta^{2}=0,$$

(33) $$\frac{\delta }{Sc}\vartheta^{\prime \prime }-H\vartheta^{\prime }+k_{1}\chi \vartheta^{2}=0.$$

where

(34) $$\varepsilon_{1}=\frac{{\left(1-\frac{\phi_{agg}}{\phi_{\max}}\right)}^{-2.5*\phi_{\max}}}{\left(1-\phi_{agg}\right)+\phi_{agg}\frac{\rho_{agg}}{\rho_{f}}},\varepsilon_{2}=\frac{1}{\left(1-\phi_{agg}\right)+\phi_{agg}\frac{\rho_{agg}}{\rho_{f}}},\varepsilon_{3}=\frac{1}{\left(1-\phi_{agg}\right)+\phi_{agg}\frac{{\left(\rho C_{p}\right)}_{agg}}{{\left(\rho C_{p}\right)}_{f}}}.$$

The modified boundary conditions for the proposed work are as follows:

(35) $$\left.\begin{array}{l} F\left(0\right)=\omega ,G\left(0\right)=1,\varepsilon_{2}\frac{k_{nf}}{k_{f}}M_{e}\theta^{\prime }\left(0\right)+\mathrm{Pr}H(0)=0,\theta \left(0\right)=0,\chi^{\prime }(0)=k_{2}\chi (0),\\ \delta \vartheta^{\prime }(0)=-k_{2}\chi (0),F\left(\infty \right)\to 0,G\left(\infty \right)\to 0,H\left(\infty \right)\to 0,\theta \left(\infty \right)\to 1,\chi \left(\infty \right)\to 1,\vartheta \left(\infty \right)\to 0. \end{array}\right\}$$

Dimensionless parameters for the proposed work are as follows:

(36) Pr=μfCpkf,k1=kca02Ω,Sc=νfDA,R=16σ∗T∞33k∗kf,ω=C0Ω,k2=ksDAνfΩ,M=σfB2ρfΩ,δ=DBDA,Re=r2Ωνf,Me=Cf(T∞−Tm)λ+Cs(Tm−T0).

The $A^{*}$ and $B^{*}$ are assumed to be of identical magnitude in this case. As a result of this logic, we must assume that the $D_{A}$ and $D_{B}$ are equivalent, i.e., δ=1 and thus

(37) χ(η)+ϑ(η)=1.

Now by using Equation (37), in Equations (32) and (33) we obtain

(38) $$\frac{1}{Sc}\chi^{\prime \prime }-H\chi^{\prime }-k_{1}\chi {\left(1-\chi \right)}^{2}=0,$$

along with the boundary constraints

(39) $$\chi^{\prime }(0)=k_{2}\chi (0),\chi (\infty )\to 1.$$

The local skin friction coefficient and the Nusselt number can be written as (see Imtiaz et al. [41]):

(40) CfRe12=1−ϕaggϕmax−2.5*ϕmaxF′(0)2+G′(0)2,

(41) NuRe−12=−knfkf+Rθ′(0).

## 3. Results and Discussions

**Figure 2:**
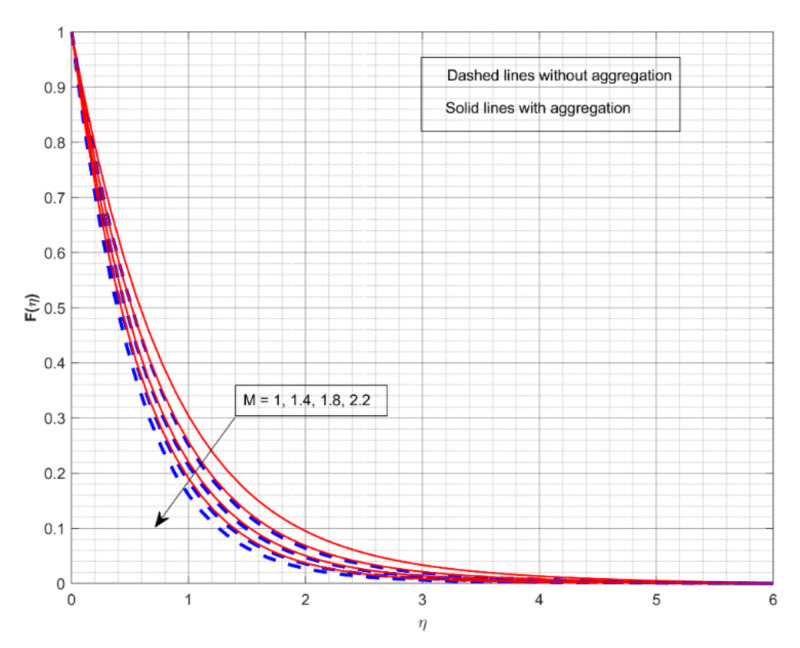
The impact of magnetic parameter on the velocity profile $F\left(\eta \right)$.

**Figure 3:**
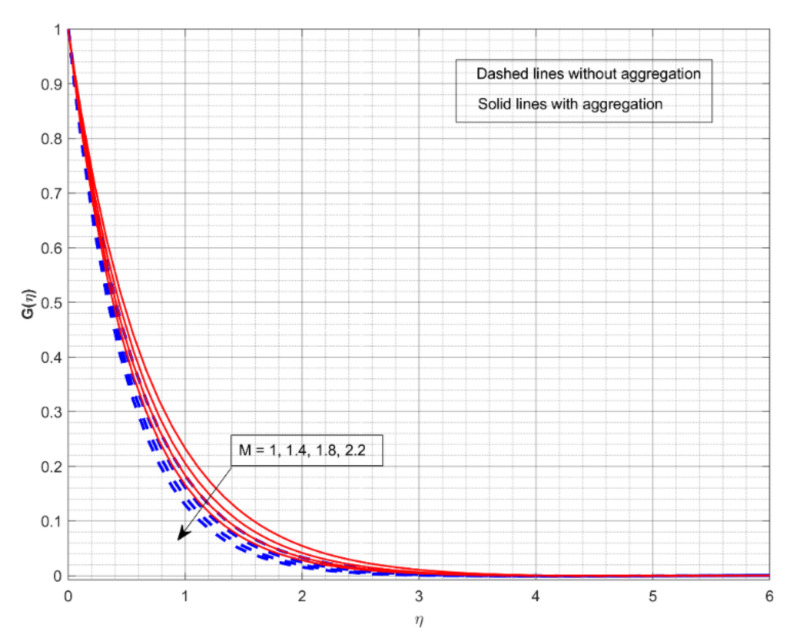
The impact of magnetic parameter on the velocity profile $G\left(\eta \right)$.

**Figure 4:**
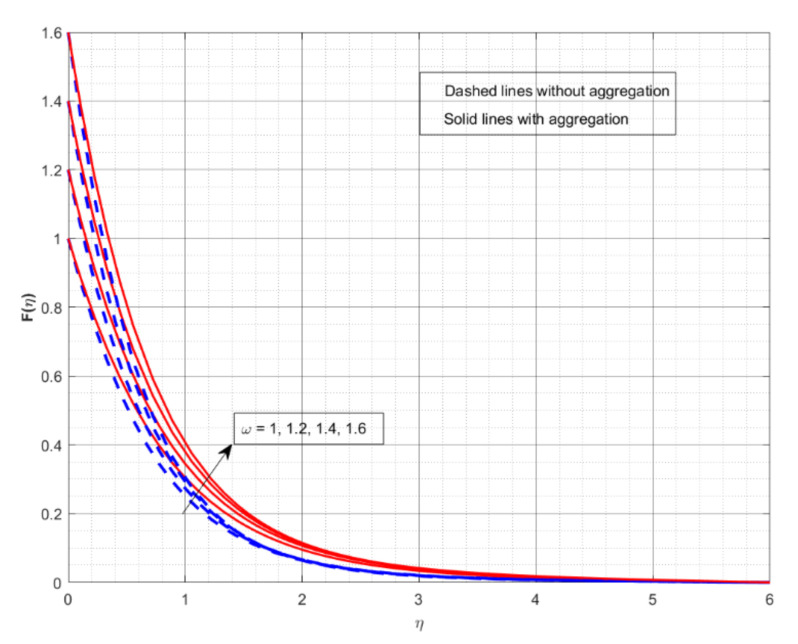
The impact of rotation-strength parameter on the velocity profile $F\left(\eta \right)$.

**Figure 5:**
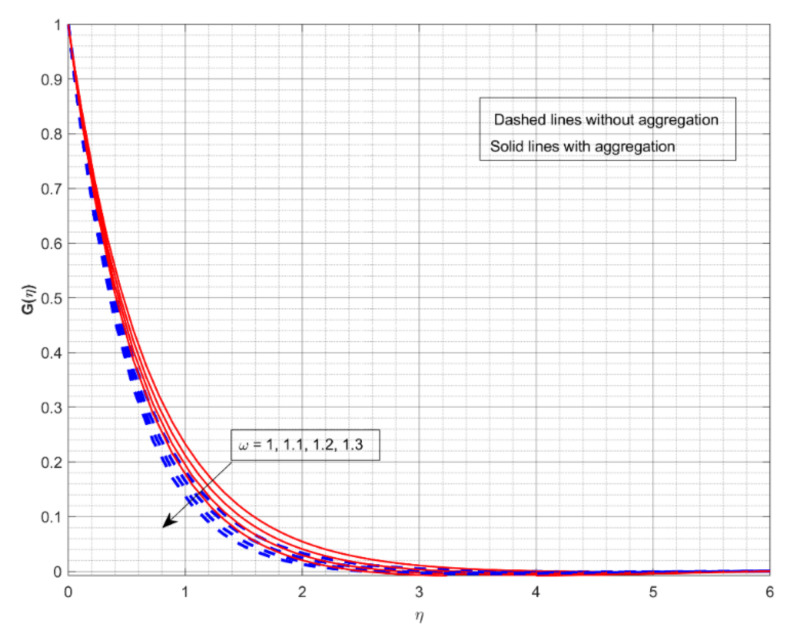
The impact of rotation-strength parameter on the velocity profile $G\left(\eta \right)$.

**Figure 6:**
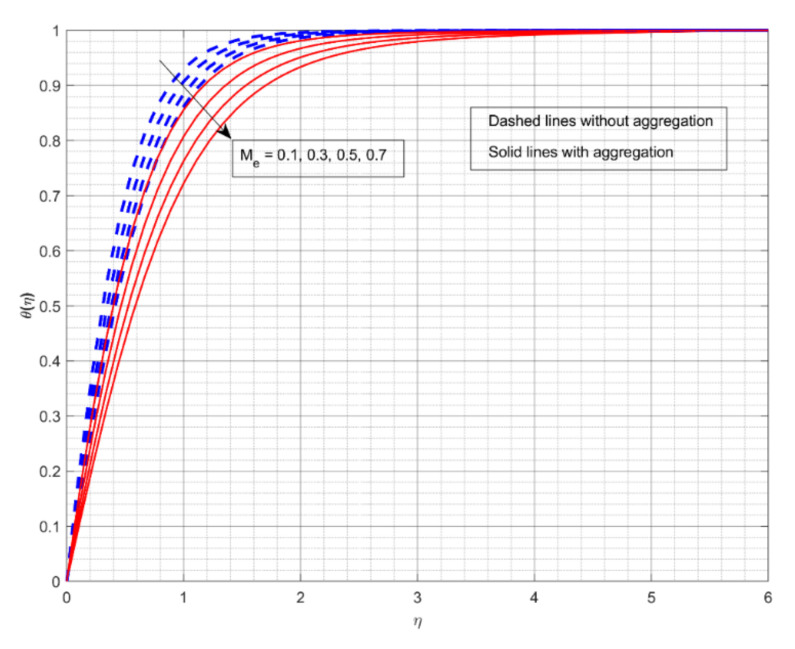
The impact of melting parameteron the thermal profile $\theta \left(\eta \right)$.

**Figure 7:**
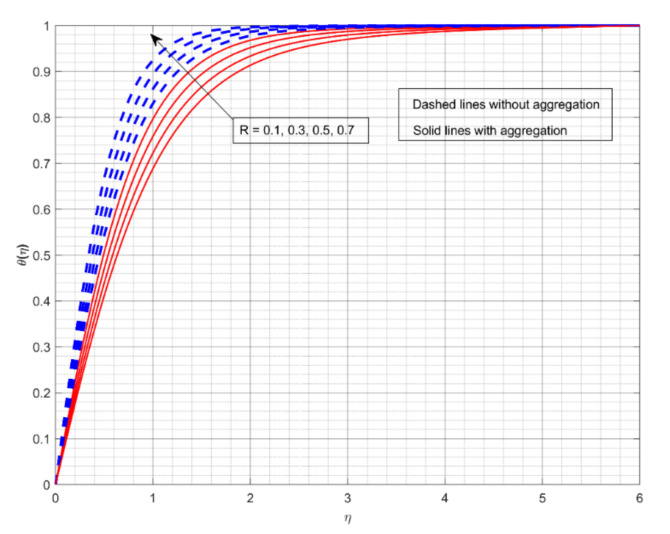
The impact of radiation parameter on the thermal profile $\theta \left(\eta \right)$.

**Figure 8:**
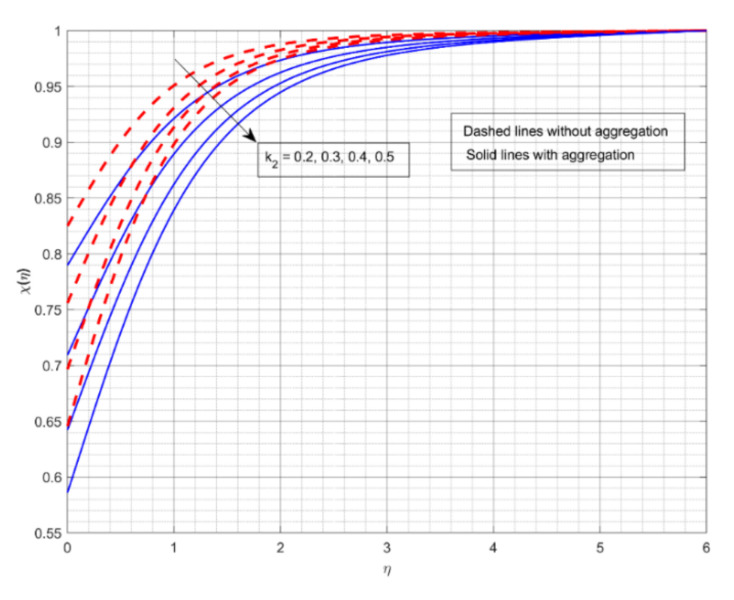
The impact of strength of heterogeneous reaction parameter on the concentration profile $\chi \left(\eta \right)$.

**Figure 9:**
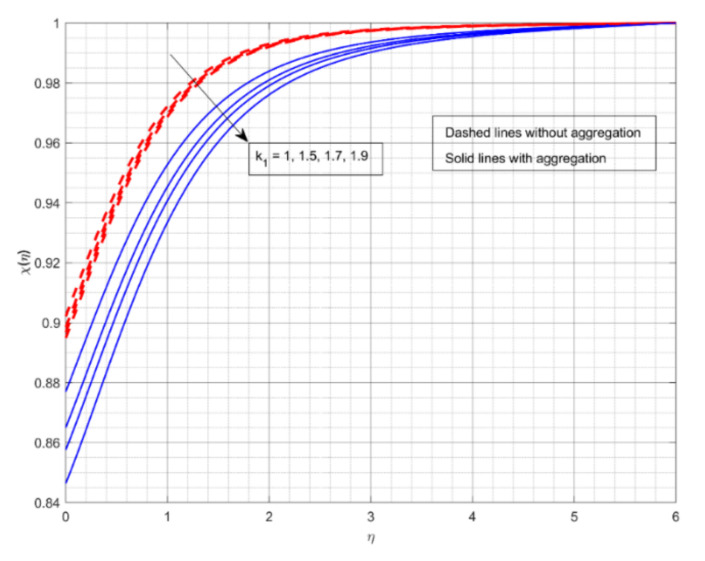
The impact of strength of homogeneous reaction parameter on the concentration profile $\chi \left(\eta \right)$.

**Figure 10:**
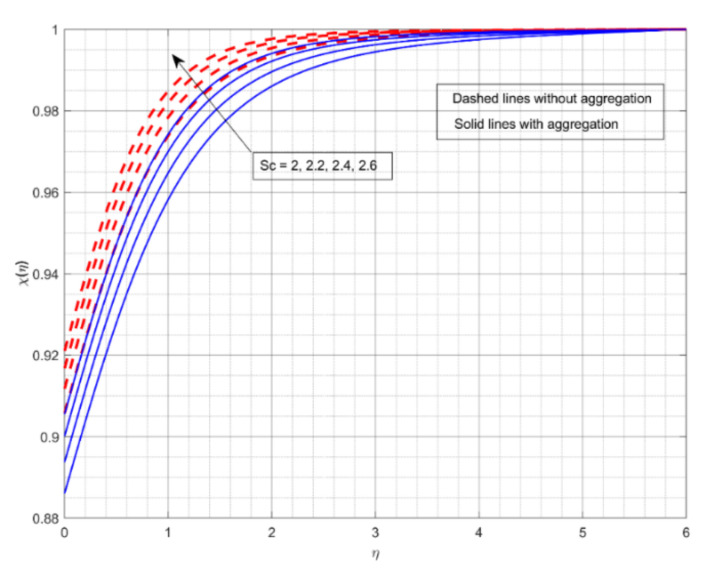
The impact of Schmidt number on the concentration profile $\chi \left(\eta \right)$.

This section displays and investigates the upshot of numerous relevant factors on respective profiles. To further understand the model’s behaviours, nonlinear ODEs are solved using the RKF-45 methodology and the shooting process. The performance of the intriguing restrictions on involved profiles is examined using graphs. This research looks at two scenarios: one with NP aggregation $\phi_{\mathrm{int}}\neq 1$ and one without aggregation $\phi_{\mathrm{int}}=1$. Flow without NP aggregation is shown in the graphs by dashed curves, but flow with NP aggregation is represented by solid lines. Physically, when more NPs are added to the base liquid, its density rises, forcing it to flow less. Further, the thermal conductivity of the nanofluid is enhanced by the attendance of more NPs. To fully verify the proposed model’s insight, we replicated the procedure with multiple parameter values. Figure 2, Figure 3, Figure 4, Figure 5, Figure 6, Figure 7, Figure 8, Figure 9 and Figure 10 show the influence of the pertinent parameters on respective profiles. The thermos-physical characteristics of the base fluid (ethylene glycol) and the nanoparticles (titanium dioxide) are written in Table 1. We also related to match the obtained numerical results with published work of Kelson and Desseaux [42], Bachok et al. [43] and Turkyilmazoglu [44] (see Table 2). From the outcomes, we have seen an excellent matching, which gives us a confidence that the obtained numerical scheme is correct and we can find the unavailable outcomes.

The effect of M on the $F\left(\eta \right)$ and $G\left(\eta \right)$ for two different cases is displayed in Figure 2 and Figure 3, respectively. The increase in M values drops down in both $F\left(\eta \right)$ and $G\left(\eta \right)$ for both cases. M is a dimensionless parameter that is used to control the velocity of the liquid. It is shown that when the magnetic field increases, the velocity profiles in all directions decrease significantly. As a result of the Lorentz force defying the motion in the system, the velocity profiles drop down with greater M values. From both figures, we conclude that the liquid velocity drops faster with NP aggregation for improved values of M. Figure 4 and Figure 5 depict the upshot of *ω* on nondimensional velocity profiles in response to various stretching ratio values. The stretching ratio is the ratio of the stretched sheet’s transverse and axial velocity. As the stretching ratio increases, the $F\left(\eta \right)$ becomes bigger than $G\left(\eta \right)$. As shown in Figure 4, a growth in the *ω* value causes the $F\left(\eta \right)$ to increase, but growth in the *ω* value declines $G\left(\eta \right)$ (see Figure 5). Here, the velocity profile improves more rapidly with NP aggregation for improved values of *ω*.

Figure 6 demonstratesthe significance of $M_{e}$ on $\theta \left(\eta \right)$. The gain in the $M_{e}$ decays the $\theta \left(\eta \right)$ for both flow cases. Here, the larger $M_{e}$ values correspond to an increased convective stream from the heated liquid to the cold surface, consequential in a reduction in heat transport. Furthermore, the liquid flow with aggregation of NPs exhibits better heat transmission than the other cases. With NP aggregation, we see less heat transference for liquid flow. Figure 7 demonstrates the upshot of R on $\theta \left(\eta \right)$. The increase in R inclines the $\theta \left(\eta \right)$ for both flow cases. Physically, increasing values of R reduces the value of the mean absorption coefficient. As a result, the radiative thermal flux indices and radiative heat-transfer rates into the fluid will improve. Additionally, the liquid flow without aggregation of NPs displays enhanced heat transport for intensified R values.

The encouragement of $k_{2}$ on $\chi \left(\eta \right)$ is shown in Figure 8. The rise in value of $k_{2}$ drops the $\chi \left(\eta \right)$. A growth in $k_{2}$ is supportive in growing the concentration of the chemical species in this case. More chemical species are likely to be involved in a chemical reaction when the rate of change in velocity for a heterogeneous reaction speeds up. Furthermore, for increased values of $k_{2}$, $\chi \left(\eta \right)$ for fluid flow without aggregation drops more slowly than for the other. With aggregation, we see minimum mass transport for the liquid stream. The upshot of $k_{1}$ on $\chi \left(\eta \right)$ is offered in Figure 9. The rise in the value of $k_{1}$ deteriorates the $\chi \left(\eta \right)$. The mass transport depreciates as the reactants are consumed throughout the homogeneous reaction. Furthermore, for increased values of $k_{1}$, $\chi \left(\eta \right)$ for liquid flow with aggregation drops sooner than for the other. Without NP aggregation, we see increased mass transfer for liquid flow. Figure 10 displays the effect of Sc on $\chi \left(\eta \right)$. The gain in Sc raises the $\chi \left(\eta \right)$. Momentum diffusivity rises as Sc rises, triggering the mass transfer to the incline. Furthermore, liquid flow without aggregation of NPs displays better mass transference than other liquid flows. 

Table 3 portrays the numerical values of the $C_{f}$ for varied ω, $M_{e}$ and M. Here, the augmented values of ω, $M_{e}$ and M improve the $C_{f}$. Moreover, improved skin friction is observed for liquid flow with NP aggregation. Table 4 displays the variation in $\theta^{\prime }(0)$ for varied R, $M_{e}$ and M. Furthermore, it is revealed from the table that the liquid stream with aggregation of NPs exhibits a higher-quality heat-passage rate for ascendant values of R, $M_{e}$ and M.

## 4. Conclusions

The present research is being conducted to investigate melting heat transfer in the presence of H-H reaction, radiation, and horizontal magnetic field effects, as well as the effects of particle aggregation on nanoliquid flow via a disc. Here, the steady incompressible flow of TiO_2_-ethylene glycol-based nanofluid is considered for comparative analysis, that is, fluid flow with and without aggregation. The new research will serve as a foundation for future stretching-flow modelling, notably in polymeric and paper production processes. The governing PDEs of the flow and heat equations are converted into ODEs with suitable similarity variables. To explain the resulting equations, the RKF-45 and shooting procedure are employed. The visual depiction of the impact of several nondimensional elements on physically interesting values. The following are the important results of the present investigation.

- The velocity profile improves faster in the case of NP aggregation for improved values of the rotation strength parameter.
- The velocity profile declines faster in the case of NP non-aggregation for improved values of the magnetic parameter.
- The liquid flow without aggregation of NPs exhibits better heat transmission for increased values of the melting parameter than the other case.
- The liquid flow without aggregation of NPs displays higher-quality heat transfer for intensified values of the radiation parameter than the remaining case.
- Mass transport declines slower for upward values of strength of the homogeneous reaction parameter for the fluid-flow case without the aggregation condition.
- Mass transport in the case of NP aggregation drops faster for cumulative values of strength of the heterogeneous reaction parameter.

## Figures and Tables

**Table 1:** Thermophysical properties for titanium dioxide and base fluid (Ethylene glycol) (see Mackolil and Mahanthesh [45]).

Properties	Titanium Dioxide	Ethylene Glycol
$\rho \left(Kg\;m^{-3}\right)$	4250	1114
$k\left(W\;m\;K^{-1}\right)$	8.9538	0.252
$\sigma \left(S\;m^{-1}\right)$	$2.38\times 10^{6}$	$1.07\times 10^{-6}$
$C_{p}\left(J\;{Kg}^{-1}\;K^{-1}\right)$	686.2	2415
$\mu \left(Kg\;m^{-1}\;s^{-1}\right)$	-	0.0157
Pr	-	204

**Table 2:** Comparison of the $F^{\prime }(0)$, $-G^{\prime }(0)$, $-H^{\prime }(\infty )$ and $-\theta^{\prime }(0)$ values for some reduced cases with available reported published work.

Comparison	$-\theta^{\prime }(0)$	$-H^{\prime }(\infty )$	$-G^{\prime }(0)$	$F^{\prime }(0)$
Kelson and Desseaux [42]	---------	0.884474	0.615922	0.510233
Bachok et al. [43]	0.9337	-----------	0.6159	0.5102
Turkyilmazoglu [44]	0.93387794	0.88447411	0.61592201	0.51023262
Present results	0.93387796	0.88447419	0.61592208	0.51023268

**Table 3:** Numerical values of the $C_{f}$ for varied ω, $M_{e}$ and M.

M	ω	$M_{e}$	$C_{f}$	
			**With NP Aggregation**	**Without NP Aggregation**
1	1	0.1	2.854255	2.2615356
1.2			3.01796	2.3703244
1.4			3.173434	2.4741029
	0.7		2.553056	1.2322237
	0.8		2.77959	1.4191719
	0.9		3.01131	1.6031328
		0.2	3.268001	2.5461544
		0.3	3.290309	2.5706962
		0.4	3.315727	2.5983268

**Table 4:** Numerical values of the $\theta^{\prime }(0)$ for varied R, $M_{e}$ and M.

M	$M_{e}$	R	Nu	
			**With NP Aggregation**	**Without NP Aggregation**
1.5	0.1	0.5	−1.626265245	−1.971101726
2			−1.66620651	−2.033848278
2.5			−1.689853329	−2.080828565
3			−1.70217352	−2.116501732
	0.2		−1.720653807	−2.074617612
	0.3		−1.829349688	−2.19135168
	0.4		−1.955731004	−2.323852014
		0.1	−1.562051004	−1.84010788
		0.2	−1.578645315	−1.877819681
		0.3	−1.594653976	−1.911974024
		0.4	−1.610474712	−1.94285506

## Data Availability

Not applicable.

## Nomenclature

E	Electric field	p	Pressure
J	Current density	Ω	Angular disk speed
$D_{A}$ and $D_{B}$	Diffusion coefficients	λ	Latent heat of the fluid
$T_{\infty }\;$	Ambient temperature	Sc	Schmidt number
R	Radiation parameter	$k^{*}$	Mean absorption coefficient
$k_{r}\;\;and\;\;k_{s}$	Rate constant	$k_{2}$	Strength of heterogeneous reaction parameter
k	Thermal conductivity	ν	Dynamic viscosity
$A^{*}$ and $B^{*}$	Chemical species	B	Magnetic field strength
ϕ	Volume fraction	$\begin{array}{l} F\left(\eta \right),G\left(\eta \right),\\ H\left(\eta \right) \end{array}$	Dimensionless velocity profiles
σ	Magnetic conductivity	M	Magnetic parameter
$P\left(\eta \right)$	Dimensionless pressure	$\theta \left(\eta \right)$	Dimensionless thermal profile
$C_{s}$	Heat capacity of the solid surface	ω	Rotation strength parameter
$C_{0}$	Constant	μ	Dynamic viscosity
$M_{e}$	Melting parameter	$k_{1}$	Strength of homogeneous reaction parameters
$T_{0}$	Solid temperature	α	Angle between the horizontal magnetic field components
(u,v,w)	Velocity components	η	Dimensionless similarity coordinate
T	Temperature	ρ	Density
Pr	Prandtl number	**Subscripts**	
$T_{m}$	Temperature of the melting surface	f	Fluid
$\sigma^{*}$	Stefan–Boltzmann constant	agg	Aggregate
(r,φ,z)	Cylindrical coordinates	nf	Nanofluid
$\rho C_{p}$	Heat capacitance	s	Solid nanoparticle
